# Supplementary material for: A panel dataset of COVID-19 vaccination policies in 185 countries
Source: Nat Hum Behav. 2023 Jul 6;7(8):1402–13. doi: 10.1038/s41562-023-01615-8 (PMC10444623; doi:10.1038/s41562-023-01615-8)
Supplement: Supplementary file 1 — Supplementary Tables 1–4. Detailed codebook description, best fit table for vaccine categories and full eliminator vs mitigator table. [file 41562_2023_1615_MOESM1_ESM.pdf]

nature human behaviour

Resource

https://doi.org/10.1038/s41562-023-01615-8

A panel dataset of COVID-19 vaccination policies in 185 countries

In the format provided by the authors and unedited

This Supplementary file includes the following:

1. Supplementary Codebook for V1-V4 indicators:
    Supplementary Table 1 Indicators V1, V2, V3, and V4, and 52 categories
    Supplementary Table 2. Additional summary indicators for indicators V1, V2, V3, and V4

2. **Supplementary Table 3**: Groups prioritised in official published plans for COVID-19 vaccination in the first- round of vaccination rollout by eliminator and mitigator countries.

3. **Supplementary 'Best Fit' Table 4** for V1-V4 indicators – to ensure standardisation for substitutions.

1. Supplementary Codebook for V1-4 indicators

Our codebook of vaccination policies contains four indicators, V1, V2, V3, and V4. For each of these we list 52 categories which were the most common groups used in vaccination rollout policies. Our full codebook is available on our Github page (https://github.com/OxCGRT/covid-policy-tracker/blob/master/documentation/codebook.md#vaccination-policies). In our full vaccines csv (https://github.com/OxCGRT/covid-policy-tracker/blob/master/data/OxCGRT_vaccines_full.csv) we publish data for each of the indicators and all 52 categories. We also publish summary indicators alongside our government policy response data (for example our 2022 national dataset here https://github.com/OxCGRT/covid-policy-tracker/blob/master/data/OxCGRT_nat_differentiated_withnotes_2022.csv). The summary indicators are automatically generated by our database, based on data entered for variables in Table 1a. These summarise the V indicators into one single value on an ordinal scale, summarising V1, V2, V2 age floor for general population, V2 age floor for at-risk population, V2 medically/clinically vulnerable categories, V2 educational categories, V2 Frontline workers (non-healthcare) categories, V2 Frontline workers (healthcare), V3, and V4.

ID	Name	Description	Measurement	Coding
**V1**	**Vaccine prioritisation**<br><br>V1_0-4 yrs Infants<br>V1_5-15 yrs Young people<br>V1_General 16-19 yrs<br>V1_General 20-24 yrs<br>V1_General 25-29 yrs<br>V1_General 30-34 yrs<br>V1_General 35-39 yrs<br>V1_General 40-44 yrs<br>V1_General 45-49 yrs<br>V1_General 50-54 yrs<br>V1_General 55-59 yrs<br>V1_General 60-64 yrs<br>V1_General 65-69 yrs<br>V1_General 70-74 yrs<br>V1_General 75-79 yrs<br>V1_General 80+ yrs<br>V1_At Risk 16-19 yrs<br>V1_At Risk 20-24 yrs<br>V1_At Risk 25-29 yrs<br>V1_At Risk 30-34 yrs<br>V1_At Risk 35-39 yrs<br>V1_At Risk 40-44 yrs<br>V1_At Risk 45-49 yrs<br>V1_At Risk 50-54 yrs<br>V1_At Risk 55-59 yrs<br>V1_At Risk 60-64 yrs<br>V1_At Risk 65-69 yrs<br>V1_At Risk 70-74 yrs<br>V1_At Risk 75-79 yrs<br>V1_At Risk 80+ yrs<br>V1_Airport/Border/Airline Staff<br>V1_Clinically vulnerable/chronic illness/significant underlying health condition (excluding elderly and disabled)<br>V1_Crowded/communal living conditions (dormitories for migrant workers, temporary	Record the ranked position for different groups within a country's prioritisation plan	Rank order	Blank – category not selected for prioritisation<br><br>1, 2, 3, 4... – category has been selected for prioritisation; number represents the rank of prioritisation; equal-ranked categories will share the same number

	accommodations)<br>V1_Disabled People<br>V1_Educators<br>V1_Ethnic minorities<br>V1_Factory workers<br>V1_Frontline/essential workers (when subcategories not specified)<br>V1_Frontline retail workers<br>V1_Healthcare workers/carers (excluding care home staff)<br>V1_Military<br>V1_Other 'high contact' professions/groups (taxi drivers, security guards)<br>V1_People living with a vulnerable/shielding person or other priority group<br>V1_Police/ first responders<br>V1_Pregnant people<br>V1_Primary and secondary school students<br>V1_Religious/Spiritual Leaders<br>V1_Residents in an elderly care home<br>V1_Staff working in an elderly care home<br>V1_Tertiary education students<br>V1_Refugees/migrants<br>V1_Government Officials			
**V2**	**Vaccine eligibility/availability**<br><br>V2_0-4 yrs Infants<br>V2_5-15 yrs Young people<br>V2_General 16-19 yrs<br>V2_General 20-24 yrs<br>V2_General 25-29 yrs<br>V2_General 30-34 yrs<br>V2_General 35-39 yrs<br>V2_General 40-44 yrs<br>V2_General 45-49 yrs<br>V2_General 50-54 yrs<br>V2_General 55-59 yrs<br>V2_General 60-64 yrs<br>V2_General 65-69 yrs<br>V2_General 70-74 yrs<br>V2_General 75-79 yrs<br>V2_General 80+ yrs<br>V2_At Risk 16-19 yrs<br>V2_At Risk 20-24 yrs<br>V2_At Risk 25-29 yrs<br>V2_At Risk 30-34 yrs<br>V2_At Risk 35-39 yrs<br>V2_At Risk 40-44 yrs<br>V2_At Risk 45-49 yrs<br>V2_At Risk 50-54 yrs<br>V2_At Risk 55-59 yrs<br>V2_At Risk 60-64 yrs<br>V2_At Risk 65-69 yrs<br>V2_At Risk 70-74 yrs<br>V2_At Risk 75-79 yrs<br>V2_At Risk 80+ yrs<br>V2_Airport/Border/Airline Staff<br>V2_Clinically vulnerable/chronic illness/significant underlying health condition (excluding elderly and disabled)<br>V2_Crowded/communal living conditions (dormitories for migrant workers, temporary accommodations)<br>V2_Disabled People<br>V2_Educators<br>V2_Ethnic minorities<br>V2_Factory workers<br>V2_Frontline/essential workers (when subcategories not specified)<br>V2_Frontline retail workers<br>V2_Healthcare workers/carers (excluding	Record which categories of people – regardless of their position in a prioritised rollout plan – are currently receiving vaccines	Categorical/ binary	Blank – no data<br><br>0 - vaccines are not being made available to this category<br><br>1 - vaccines are being made available to this category

	care home staff)<br>V2_Military<br>V2_Other 'high contact' professions/groups (taxi drivers, security guards)<br>V2_People living with a vulnerable/shielding person or other priority group<br>V2_Police/ first responders<br>V2_Pregnant people<br>V2_Primary and secondary school students<br>V2_Religious/Spiritual Leaders<br>V2_Residents in an elderly care home<br>V2_Staff working in an elderly care home<br>V2_Tertiary education students<br>V2_Refugees/migrants<br>V2_Government Officials			
**V3**	**Vaccine financial support**<br>V3_0-4 yrs Infants<br>V3_5-15 yrs Young people<br>V3_General 16-19 yrs<br>V3_General 20-24 yrs<br>V3_General 25-29 yrs<br>V3_General 30-34 yrs<br>V3_General 35-39 yrs<br>V3_General 40-44 yrs<br>V3_General 45-49 yrs<br>V3_General 50-54 yrs<br>V3_General 55-59 yrs<br>V3_General 60-64 yrs<br>V3_General 65-69 yrs<br>V3_General 70-74 yrs<br>V3_General 75-79 yrs<br>V3_General 80+ yrs<br>V3_At Risk 16-19 yrs<br>V3_At Risk 20-24 yrs<br>V3_At Risk 25-29 yrs<br>V3_At Risk 30-34 yrs<br>V3_At Risk 35-39 yrs<br>V3_At Risk 40-44 yrs<br>V3_At Risk 45-49 yrs<br>V3_At Risk 50-54 yrs<br>V3_At Risk 55-59 yrs<br>V3_At Risk 60-64 yrs<br>V3_At Risk 65-69 yrs<br>V3_At Risk 70-74 yrs<br>V3_At Risk 75-79 yrs<br>V3_At Risk 80+ yrs<br>V3_Airport/Border/Airline Staff<br>V3_Clinically vulnerable/chronic illness/significant underlying health condition (excluding elderly and disabled)<br>V3_Crowded/communal living conditions (dormitories for migrant workers, temporary accommodations)<br>V3_Disabled People<br>V3_Educators<br>V3_Ethnic minorities<br>V3_Factory workers<br>V3_Frontline/essential workers (when subcategories not specified)<br>V3_Frontline retail workers<br>V3_Healthcare workers/carers (excluding care home staff)<br>V3_Military<br>V3_Other 'high contact' professions/groups (taxi drivers, security guards)<br>V3_People living with a vulnerable/shielding person or other priority group<br>V3_Police/ first responders<br>V3_Pregnant people<br>V3_Primary and secondary school	Record how vaccines are funded for each category of people identified in V2 as currently receiving vaccines.	Ordinal scale	Blank - no data<br><br>1 - full cost borne by the individual (or through private health insurance) or no policy<br><br>2 - partially funded by government and individual pays nominal fee<br><br>3 - fully covered by government funding, FREE

	students<br>V3_Religious/Spiritual Leaders<br>V3_Residents in an elderly care home<br>V3_Staff working in an elderly care home<br>V3_Tertiary education students<br>V3_Refugees/migrants<br>V3_Government Officials			
**V4**	**V4_Vaccine requirement/mandate**<br><br>V4_0-4 yrs Infants<br>V4_5-15 yrs Young people<br>V4_General 16-19 yrs<br>VV4_General 20-24 yrs<br>V4_General 25-29 yrs<br>V4_General 30-34 yrs<br>V4_General 35-39 yrs<br>V4_General 40-44 yrs<br>V4_General 45-49 yrs<br>V4_General 50-54 yrs<br>V4_General 55-59 yrs<br>V4_General 60-64 yrs<br>V4_General 65-69 yrs<br>V4_General 70-74 yrs<br>V4_General 75-79 yrs<br>V4_General 80+ yrs<br>V4_At Risk 16-19 yrs<br>V4_At Risk 20-24 yrs<br>V4_At Risk 25-29 yrs<br>V4_At Risk 30-34 yrs<br>V4_At Risk 35-39 yrs<br>V4_At Risk 40-44 yrs<br>VV4_At Risk 45-49 yrs<br>V4_At Risk 50-54 yrs<br>V4_At Risk 55-59 yrs<br>V4_At Risk 60-64 yrs<br>V4_At Risk 65-69 yrs<br>V4_At Risk 70-74 yrs<br>V4_At Risk 75-79 yrs<br>V4_At Risk 80+ yrs<br>V4_Airport/Border/Airline Staff<br>V4_Clinically vulnerable/chronic illness/significant underlying health condition (excluding elderly and disabled)<br>V4_Crowded/communal living conditions (dormitories for migrant workers, temporary accommodations)<br>V4_Disabled People<br>V4_Educators<br>V4_Ethnic minorities<br>V4_Factory workers<br>V4_Frontline/essential workers (when subcategories not specified)<br>V4_Frontline retail workers<br>V4_Healthcare workers/carers (excluding care home staff)<br>V4_Military<br>V4_Other 'high contact' professions/groups (taxi drivers, security guards)<br>V4_People living with a vulnerable/shielding person or other priority group<br>V4_Police/ first responders<br>V4_Pregnant people<br>V4_Primary and secondary school students<br>V4_Religious/Spiritual Leaders<br>V4_Residents in an elderly care home<br>V4_Staff working in an elderly care home<br>V4_Tertiary education students<br>V4_Refugees/migrants<br>V4_Government Officials	Reports the existence of a requirement to be vaccinated	Binary	Blank - no data<br><br>0 - no requirement to be vaccinated<br><br>1 - requirement to be vaccinated

Supplementary Table 1. Indicators V1, V2, V3, and V4, and 53 categories.

ID	Name	Description	Measurement	Coding
V1	V1_Vaccine prioritisation (summary)	Reports the existence of a prioritised plan for vaccine rollout	Ordinal scale	Blank – no data<br>0 - no plan<br>1 – a prioritised plan is in place<br>2 – universal/general eligibility; no prioritisation between groups
V2A	V2_Vaccine eligibility/availability (summary)	Reports whether any categories of people are receiving vaccines	Ordinal scale	Blank – no data<br>0 – no categories are receiving vaccines<br>1 – vaccines are available to some categories<br>2 – vaccines are available to anyone over the age of 16 yrs<br>3 – vaccines are available to anyone over the age of 16 yrs PLUS one or both of 5-15 yrs and 0-4 yrs
V2B	V2B_Vaccine age eligibility/availability age floor(General population summary)	Reports lowest age range of general population being vaccinated	Numerical	Blank – no data<br>0 – no categories are receiving vaccines<br>numerical range – Lowest age range for 'General' category
V2C	V2C_Vaccine age eligibility/availability age floor(At-risk population summary)	Reports lowest age range of at risk population being vaccinated	Numerical	Blank – no data<br>0 – no categories are receiving vaccines<br>numerical range – Lowest age range from either 'General' or 'At-risk' categories
V2D	V2D_Medically/ clinically vulnerable (Non-elderly)	Reports the number of categories selected from thematic group:<br>V2_At risk age ranges below 60 (one or more selected counts as 1 x category)<br>V2_Clinically vulnerable/chronic illness/significant underlying health condition (excluding elderly and disabled)<br>V2_Disabled people<br>V2_Pregnant people<br>V2_People living with a vulnerable/shielding person or other priority group	Ordinal	Blank – no data<br>0 – no categories are receiving vaccines<br>1 – 1 or 2 categories in group selected<br>2 – 3 or more categories selected or all from V2_General 16-19 years up to V2_General 80+ years present
V2E	V2E_Education	Reports the number of categories selected from thematic group:<br>V2_Educators<br>V2_Primary and secondary school students<br>V2_Tertiary education students	Ordinal	Blank – no data<br>0 – no categories are receiving vaccines<br>1 – 1 category in group selected<br>2 - 2 or more categories selected or all from V2_General 16-19 years up to V2_General 80+ years present
V2F	V2F_Frontline workers (non-healthcare)	Reports the number of categories selected from thematic group:<br>V2_Police/first responders<br>V2_Airport/Border/Airline staff<br>V2_Factory workers<br>V2_Frontline retail workers<br>V2_Military<br>V2_Other high contact professions/groups (taxi drivers, security guards)<br>V2_Frontline/essential workers (when subcategories not specified) (triggers an automatic 2)	Ordinal	Blank – no data<br>0 – no categories are receiving vaccines<br>1 – 1 or 2 categories in group selected<br>2 - 3 or more categories selected or all from V2_General 16-19 years up to V2_General 80+ years present
V2G	V2F_Frontlin	Reports the number of categories	Ordinal	Blank – no data

	e workers (healthcare)	selected from thematic group:<br>V2_Staff working in an elderly care home<br>V2_Healthcare workers/carers (excluding care home staff)		0 – no categories are receiving vaccines<br>1 – 1 category in group selected<br>2 - 2 categories selected or all from V2_General 16-19 years up to V2_General 80+ years present
V3	V3_Vaccine financial support (summary)	Reports the overall approach taken to vaccine funding – whether paid by the individual or the government	Ordinal scale	Blank - no data<br>0 – no availability<br>1 – full cost to the individual for all categories identified in V2<br>2 – full cost to the individual for some categories identified in V2, some subsidy for other categories<br>3- partial funding by the government for all of the categories identified in V2<br>4 – partial funding by the government for some categories identified in V2, full funding for other categories<br>5 – all categories fully funded by the government
V4	V4_Mandatory Vaccination (summary)	Reports the existence of a requirement to be vaccinated	Binary	Blank - no data<br>0 – no requirement to be vaccinated<br>1 – requirement to be vaccinated is in place for one or more groups

Supplementary Table 2. Additional summary indicators for indicators V1, V2, V3, and V4.

3.Supplementary ‘Best Fit’ Table for V1-4 indicators

Oftentimes there is a vague or ambiguous reference to a group of people, or a category listed on a policy document that is not in our existing list. In these circumstances our data collectors use the below ‘Best Fit Table’ to select the category which best represents this, which is available on Github (https://github.com/OxCGRT/covid-policy-tracker/blob/master/documentation/interpretation_guide.md). This ensures a standardization of ‘best fit’ category selection where categories have been substituted. Qualitative coding notes can be used to extract country-specific categories for further analysis.

OxCGRT Category	Examples of country-designated categories that have resulted in this box being ticked
Police/ first responders	Occupations important to functioning of society (IRL)<br>Groups of persons who are of critical importance to the functioning of Singapore (SGP)<br>Ambulance and paramedic staff (AUS)<br>Emergency health staff (FIN)<br>Firefighters (FRA)<br>Fire (AUS)
Disabled people	People with a learning or neurological disability (GBR)<br>People with Down’s Syndrome (PRT)<br>People in communal facilities with an increased risk of infection and outbreaks (for example homes for the handicapped) (CHE)
Border staff	Key workers in essential jobs who cannot avoid high risk of exposure (IRL)<br>Maritime and aviation (SGP)
Frontline retail workers	Restaurant workers
Frontline workers (when not otherwise specified)	Other people aged 65-69 and key workers essential to the vaccine programme’ (IRL)<br>Workers identified as performing a critical function in society [unspecified] (DNK)<br>Operators essential for the country's economic activities (FRA)<br>Essential professions in this phase, people with essential social and/or economic profession are vaccinated (BEL)
Tertiary education students	University, college, or technical trade schools
Educators	University, college, or technical trade schools<br>Teachers in any level of school<br>Instructors/professors in colleges and universities

Other high contact professions	Disability care staff (AUS)<br>Olympic/professional/international athletes (BRB)
Ethnic minorities	Aboriginal and Torres Strait Islander people > 55 (AUS)<br>Indigenous populations (CAN)
Factory staff	Meat processing staff (AUS)
Crowded/communal living conditions (dormitories for migrant workers, temporary accommodations)	People in communal facilities with an increased risk of infection and outbreaks (with residents of mixed ages) (SGP)<br>People living or working in crowded settings (IRL)<br>Prison populations (ISR)<br>People who live in socially vulnerable situations, such as the homeless or the undocumented. (SWE)<br>Homeless (KOR)<br>Vulnerable and precarious people (homeless…), living in communities (prisons, psychiatric establishments, homes) (FRA)<br>People in communal facilities with an increased risk of infection and outbreaks (with residents of mixed ages) (CHE)

Supplementary Table 4. A 'Best Fit' table for categories not on the list to ensure standardisation for substitutions.
