# Supplementary material for: A panel dataset of COVID-19 vaccination policies in 185 countries
Source: Nat Hum Behav. 2023 Jul 6;7(8):1402–13. doi: 10.1038/s41562-023-01615-8 (PMC10444623; doi:10.1038/s41562-023-01615-8)
Supplement: Supplementary file 2 — Reporting Summary [file 41562_2023_1615_MOESM2_ESM.pdf]

nature portfolio

Corresponding author(s): Emily Cameron-Blake

Last updated by author(s): Apr 18, 2023

Reporting Summary

Nature Portfolio wishes to improve the reproducibility of the work that we publish. This form provides structure for consistency and transparency in reporting. For further information on Nature Portfolio policies, see our Editorial Policies and the Editorial Policy Checklist.

Statistics

For all statistical analyses, confirm that the following items are present in the figure legend, table legend, main text, or Methods section.

n/a	Confirmed	
☐	☒	The exact sample size (n) for each experimental group/condition, given as a discrete number and unit of measurement
☐	☒	A statement on whether measurements were taken from distinct samples or whether the same sample was measured repeatedly
☒	☐	The statistical test(s) used AND whether they are one- or two-sided<br>*Only common tests should be described solely by name; describe more complex techniques in the Methods section.*
☐	☒	A description of all covariates tested
☒	☐	A description of any assumptions or corrections, such as tests of normality and adjustment for multiple comparisons
☒	☐	A full description of the statistical parameters including central tendency (e.g. means) or other basic estimates (e.g. regression coefficient) AND variation (e.g. standard deviation) or associated estimates of uncertainty (e.g. confidence intervals)
☒	☐	For null hypothesis testing, the test statistic (e.g. F, t, r) with confidence intervals, effect sizes, degrees of freedom and P value noted<br>*Give P values as exact values whenever suitable.*
☒	☐	For Bayesian analysis, information on the choice of priors and Markov chain Monte Carlo settings
☒	☐	For hierarchical and complex designs, identification of the appropriate level for tests and full reporting of outcomes
☒	☐	Estimates of effect sizes (e.g. Cohen's d, Pearson's r), indicating how they were calculated

Our web collection on statistics for biologists contains articles on many of the points above.

Software and code

Policy information about availability of computer code

Data collection	Google translate (online web version, http://translate.google.com) was used in the data collection process to aid translation and understanding of data for collection.
Data analysis	Stata (V17.0), Microsoft Excel (V 16) and R (4.1.3) were used to manipulate and analyse the data for visualisations and Figures in this article.

For manuscripts utilizing custom algorithms or software that are central to the research but not yet described in published literature, software must be made available to editors and reviewers. We strongly encourage code deposition in a community repository (e.g. GitHub). See the Nature Portfolio guidelines for submitting code & software for further information.

Data

Policy information about availability of data

All manuscripts must include a data availability statement. This statement should provide the following information, where applicable:

- Accession codes, unique identifiers, or web links for publicly available datasets
- A description of any restrictions on data availability
- For clinical datasets or third party data, please ensure that the statement adheres to our policy

The vaccine policy data are available on GitHub (use the download tab to download linked file): https://github.com/OxCGRT/covid-policy-tracker/blob/master/data/OxCGRT_vaccines_full.csv . All other information (including methodology and documentation) is available at: https://github.com/OxCGRT

There are no restrictions on data availability.

Human research participants

Policy information about studies involving human research participants and Sex and Gender in Research.

Reporting on sex and gender	N/A
Population characteristics	N/A
Recruitment	N/A
Ethics oversight	N/A

Note that full information on the approval of the study protocol must also be provided in the manuscript.

Field-specific reporting

Please select the one below that is the best fit for your research. If you are not sure, read the appropriate sections before making your selection.

☐ Life sciences ☒ Behavioural & social sciences ☐ Ecological, evolutionary & environmental sciences

For a reference copy of the document with all sections, see nature.com/documents/nr-reporting-summary-flat.pdf

Behavioural & social sciences study design

All studies must disclose on these points even when the disclosure is negative.

Study description

This 'resource' article describes and presents a new dataset that provides quantitaive and qualitative data on COVID-19 vaccination policies around the globe. It does not carry out causal inference.

Research sample

The new data and database presented in this article includes information on most countries in the world (185), as well as a number of subnational jurisdictions in the United States, Canada, United Kingdom and China. Countries were included in the dataset due to the ease/ability to collect the relevant data, and volunteer data collector capacity. The sample of countries in this dataset is representative of the global population and of each continent.

Sampling strategy

Sampling is not relied upon for this article. This article profiles a newly developed dataset of COVID-19 vaccine policies, and the countries therein were included based on the ability to find and record relevant policy data. Sufficient countries (185) are included in this dataset to enable cross-country comparisons.

Data collection

The OxCGRT database is maintained by a large team of specially trained volunteer data contributors from around the globe. Initially volunteers were recruited in March of 2020 largely from the postgraduate student body of the Blavatnik School of Government at the University of Oxford. Since then, additional contributors have been recruited through Oxford University departmental mailing lists, student societies and alumni lists as well as through social media channels of other large university networks and via a contributor group on LinkedIn. Many volunteers have been referred by existing or previous volunteers. To date, the OxCGRT has had over 1500 specially trained data-contributors.

New members of the data collection tam undergo a series of training steps. First, they complete a bespoke e-learning course (designed by lead author E.CB.) that describes which COVID-19 non-pharmaceutical intervention (NPI) and vaccine policies the OxCGRT collects data on, how our ordinal/binary/categorical indicators are coded in the database, and how the database operates and how to contribute data. The e-learning course also includes practice questions and a test that must be passed with a minimum score of 80% in order to progress. The test assesses comprehension and understanding of the coding schema and collection process. After this training process, contributors are assigned to various 'teams' within the OxCGRT based on local knowledge, language, and needs of the program, and are assigned weekly tasks for updating data. All data collectors are expected to attend weekly contributor meetings where they are able to ask questions of the core OxCGRT team for clarity, if needed. For the vaccine policies, it was not possible to create an endless list of categories of people/groups, so a 'best-fit' table has been created and is available to both contributors and data users on GitHub.

OxCGRT collects national data on a weekly schedule, during which new task allocations are sent to the data collection team. For the addition of the vaccine policy dataset, experienced contributors were recruited to an initial team to begin building the data in the database from March 2021. Once each country/jurisdcition was 'built' and updated with all vaccine policies, the allocation of updates to the vaccine policies was added into the regular weekly rotation of updating data for the OxCGRT. The data is published in real-time as contributors enter it into the system.

Once data is entered into the database, it is marked as 'provisional', which flags it for a review process. First, after each allocation round, a small team will do quick spot checks to ensure that data has been entered properly and there are no gross errors. The provisional data is then queued for attention by a more thorough review team. Initial reviews for this dataset were completed by

lead authors E.CB. and H.T., then by specially trained reviewers on the vaccine policies (trained by a bespoke e-learning course). Initial reviews suggest a high degree of accuracy in the initial data collection (90% of initial entries were correct, the other 10% were corrected - often a simple date change or inclusion/exclusion of a particular group for vaccination).

Data is collected from publicly available sources such as government press releases and briefings, international organisation reports, and trusted news articles. Original source materials are archived and saved as notes within the database so that coding can be checked and substantiated.

For this article, there was no study hypotheis required/developed, nor a requirement to be blinded to experimental conditions.

Timing	Collection of the vaccine policy data began in March 2021 and continues through the present. All data from January 1 2020 until 31 December 2022 are recorded in the database.
Data exclusions	This article contains no in-depth analysis, but presents 'snapshots' of the data to demonstrate potential uses and trends. No data are excluded from these presentations.
Non-participation	There are no participants in this study
Randomization	This study/article does not rely on randomization as it is presenting a new dataset, and some insights for motivation of future use of the dataset.

Reporting for specific materials, systems and methods

We require information from authors about some types of materials, experimental systems and methods used in many studies. Here, indicate whether each material, system or method listed is relevant to your study. If you are not sure if a list item applies to your research, read the appropriate section before selecting a response.

Materials & experimental systems

n/a	Involved in the study	
☒	☐	Antibodies
☒	☐	Eukaryotic cell lines
☒	☐	Palaeontology and archaeology
☒	☐	Animals and other organisms
☒	☐	Clinical data
☒	☐	Dual use research of concern

Methods

n/a	Involved in the study	
☒	☐	ChIP-seq
☒	☐	Flow cytometry
☒	☐	MRI-based neuroimaging
